# Influence of cell physiological state on gene delivery to T lymphocytes by chimeric adenovirus Ad5F35

**DOI:** 10.1038/srep22688

**Published:** 2016-03-14

**Authors:** Wen-feng Zhang, Hong-wei Shao, Feng-lin Wu, Xin Xie, Zhu-Ming Li, Hua-Ben Bo, Han Shen, Teng Wang, Shu-lin Huang

**Affiliations:** 1 Guangdong Province Key Laboratory for Biotechnology Drug Candidates, Guang dong Pharmaceutical University, Guang zhou, People’s Republic of China; 2 School of Biosciences and Biopharmaceutics, Guangdong Pharmaceutical University, Guang zhou, People’s Republic of China

## Abstract

Adoptive transfer of genetically-modified T cells is a promising approach for treatment of both human malignancies and viral infections. Due to its ability to efficiently infect lymphocytes, the chimeric adenovirus Ad5F35 is potentially useful as an immunotherapeutic for the genetic modification of T cells. In previous studies, it was found that the infection efficiency of Ad5F35 was significantly increased without enhanced expression of the viral receptor after T cell stimulation; however, little is known about the underlying mechanism. Nonetheless, cell physiology has long been thought to affect viral infection. Therefore, we aimed to uncover the physiologic changes responsible for the increased infection efficiency of Ad5F35 following T cell stimulation. Given the complexity of intracellular transport we analyzed viral binding, entry, and escape using a Jurkat T cell model and found that both cell membrane fluidity and endosomal escape of Ad5F35 were altered under different physiological states. This, in turn, resulted in differences in the amount of virus entering cells and reaching the cytoplasm. These results provide additional insight into the molecular mechanisms underlying Ad5F35 infection of T cells and consequently, will help further the clinical application of genetically-modified T cells for immunotherapy.

Adenoviruses (Ads) have a defined genetic background, high titers, and low pathogenicity. Notably, Ads do not integrate into the host genome and can transfect both dividing and non-dividing cells *in vivo*. Given these advantages, these viruses are extensively used as viral vectors in clinical gene therapy trials^1^,^2^. Over 100 different wild-type Ads have been isolated. Among these, the well-characterized Serotype 5 adenovirus (Ad5) is currently the most commonly used adenoviral vector for gene therapy^3^. Ad5 recognizes the coxsackie receptor (CAR) as a natural cell surface receptor; however, due to the low expression of CAR on T lymphocytes, the infection efficiency of Ad5 is relatively low, limiting its use in the genetic modification of T cells. Adenovirus type 35 (Ad35) is a subgroup B adenovirus that recognizes CD46, which is highly expressed on human CD3^+^ T cells, as its natural cellular receptor^4^. Thus, given that Ad35 is relatively uncharacterized, replacement of the fiber gene in adenovirus type 5 with the Ad35 fiber gene (in order to generate the chimeric adenovirus vector Ad5F35) improves the T cell infection efficiency and increases the therapeutic potential of this virus for use in adoptive immunotherapy with genetically-modified T cells^4^.

In a previous study, we found that the efficiency of Ad5F35 infection of human peripheral blood mononuclear cells (PBMCs) is significantly improved following *in vitro* stimulation with OKT3 and cytokines. In addition, the efficiency of viral infection is thought to be affected mainly by the expression of viral receptors. For example, Seidman *et al.* found that Ad5 is better able to infect dividing cells than non-dividing cells due to the fact that cells undergoing division express more viral receptors than do non-dividing cells^5^. However, we and others found no significant changes in the expression of the Ad5F35 receptor, CD46, after T cell stimulation^4^. There is evidence that the physiological state of a cell can also affect the efficiency of viral infection^6^,^7^. To this end, we show that stimulation of T cells results in significant changes in their physiological state and increased Ad5F35 infection efficiency without significant changes in viral receptor expression. Therefore, factors besides receptor expression affect the efficiency of Ad5F35 infection of T cells.

Thus, we aimed to uncover the physiologic changes in T cells responsible for the increased Ad5F35 infection efficiency. Because PBMCs are heterogeneous and the purification of T cells inevitably results in their stimulation, we selected the Jurkat (leukemic T cell line) for use as a model in our experiments. Given the activated status of Jurkat cells, the serum starvation method was used to inversely induce changes in the physiological state of Jurkat cells, which successfully replicated the observed *in vitro* phenomenon of altered efficiency of viral infection without changes in the membrane expression of viral entry receptors. We analyzed viral binding, entry, and escape of Ad5F35 and found that changes to the physiological state of T cells can significantly alter the processes of viral entry and escape. These results form the foundation for use of this viral vector for adoptive immunotherapy based on the genetic modification of T cells.

## Results

### Cell surface receptor expression is not the only factor affecting the efficiency of Ad5F35 infection of T cells

**Figure 1:**
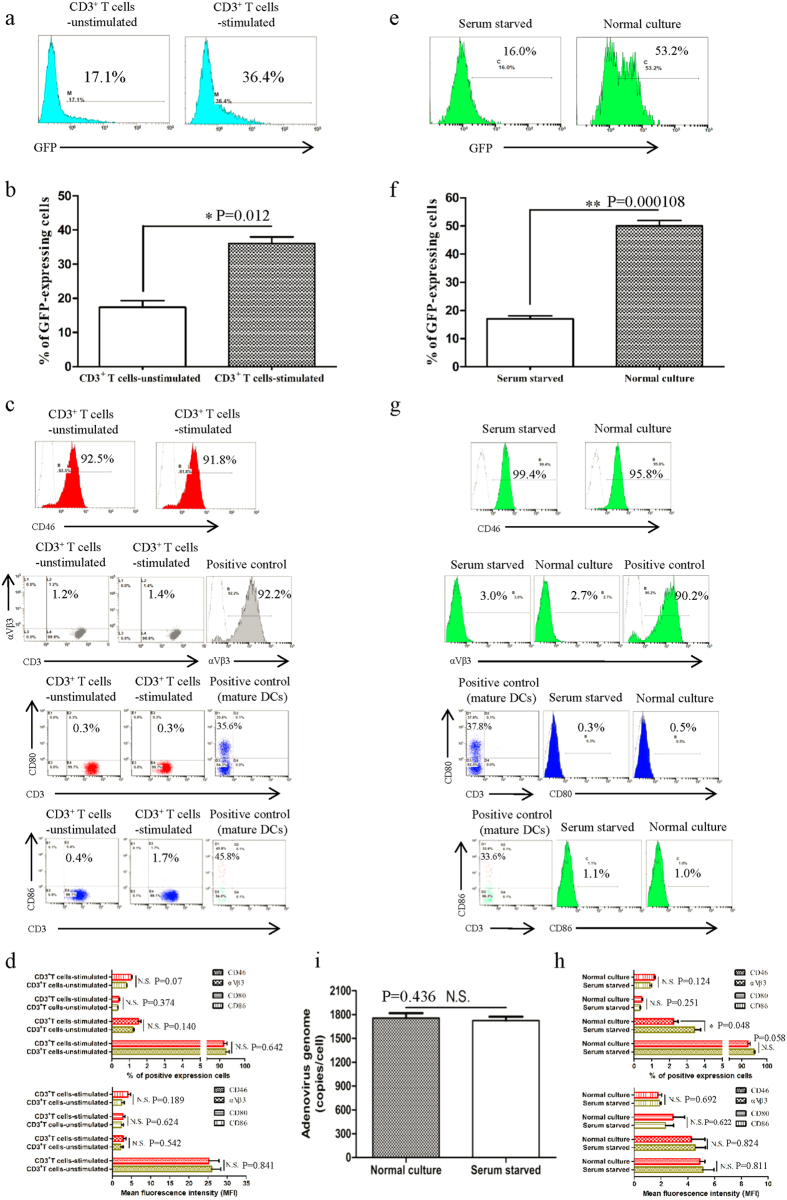
Cell surface receptor expression is not the only factor affecting the efficiency of Ad5F35 infection of T cells. (**a**,**b**) PBMCS were infected with Ad5F35-GFP before and after stimulation with OKT3 and cytokines. The percentage of GFP-positive T lymphocytes was determined by flow cytometry at 48 h after infection. The data are presented as the mean ± standard error of three experiments. (*p < 0.05). (**c**,**d**) Antibodies against CD46, integrin αVβ3, CD80, and CD86 were incubated with PBMCs before and after stimulation. Both the percentage of receptor positive cells and the MFI among T lymphocytes were determined by flow cytometry. The data are presented as the mean ± standard error of three experiments. (N.S. no statistical significance). (**e**,**f**) After 72 h of normal culture or serum starvation, Jurkat cells were infected with Ad5F35-GFP and the percentage of GFP-positive cells was determined by flow cytometry. The data are presented as the mean ± standard error of three experiments. (**p < 0.01). (**g**,**h**) After 72 h of normal culture or serum starvation, Jurkat cells were incubated with CD46, integrin αVβ3, CD80, and CD86 antibodies, both the percentage of receptor positive cells and the MFI were determined by flow cytometry. The data are presented as the mean ± standard error of three experiments. (N.S. no statistical significance). (**i**) After 72 h of normal culture or serum starvation, Ad5F35-GFP was added to Jurkat cells at an MOI of 100 and incubated on ice for 1 h. The supernatant was discarded, and the remainder was washed three times with phosphate-buffered saline (PBS). Total genomic DNA was extracted and the mean amount of virus bound per cell was measured by fluorescence quantitative PCR. The data are presented as the mean ± standard error of three experiments. (N.S. no statistical significance).

T cell lines and primary T cells were infected with Ad5F35-GFP virus and GFP expression was analyzed 48 h post-transduction by flow cytometry. We observed a greater than two-fold increase in the infection efficiency of Ad5F35 into PBMCs following stimulation (Fig. 1a,b) (Independent-Samples T Test; t = 4.316; p-value = 0.012). Next, we analyzed the changes in expression of CD46 and integrin αVβ3 after stimulation and observed no significant changes in the surface expression of either (Fig. 1c,d). Interactions between Ad5F35 and CD80 and CD86 have been reported and as a result, these two molecules have been proposed to act as novel receptors for this virus^8^. To this end, we assessed the expression of CD80 and CD86 on T cells after stimulation and found there to be no difference from that seen on resting cells (Fig. 1c,d).

Given the heterogeneity of PBMCs, we used Jurkat T cells in subsequent experiments in order to facilitate the study of the mechanism by which Ad5F35 infects T cells. Given the active status of Jurkat cells, serum starvation method was used to inversely induce the changes in the physiological state of Jurkat cells. Jurkat cells were either left untreated or were cultured under serum starvation (medium containing only 1% serum) for 72 hours followed by infection with Ad5F35. The results showed a significant reduction in the infection efficiency of Ad5F35 following serum limitation (from 50% to 17%; Fig. 1e,f) (Independent-Samples T Test; t = 15.257; p-value = 0.000108). Notably, consistent with experiments done with PBMCs, expression levels of CD46, integrin αVβ3, CD80, or CD86 did not change significantly (Fig. 1g,h). We next analyzed the amount of cell-bound virus before and after serum starvation. To this end, cells and virus were co-incubated in an ice bath for 1 hour to allow for viral binding to cells but to inhibit viral entry via endocytosis. After incubation, the cellular and viral genomes were extracted and used for real-time PCR analysis. Our results show that the amount of cell-bound virus in the untreated culture group was 1,756 ± 24.5 copies/cell, while the amount of cell-bound virus in the serum starvation group was 1,723 ± 29.2 copies/cell (Fig. 1i). Thus, there was no significant difference between the two cultures, indicating that serum starvation does not affect viral binding to cells. Taken together, our results indicate that the alteration of virus-binding capacity resulting from changes in receptor expression levels is not the only factor affecting the efficiency of Ad5F35 infection of T cells.

### The infection efficiency of Ad5F35 is associated with virus endocytosis

**Figure 2:**
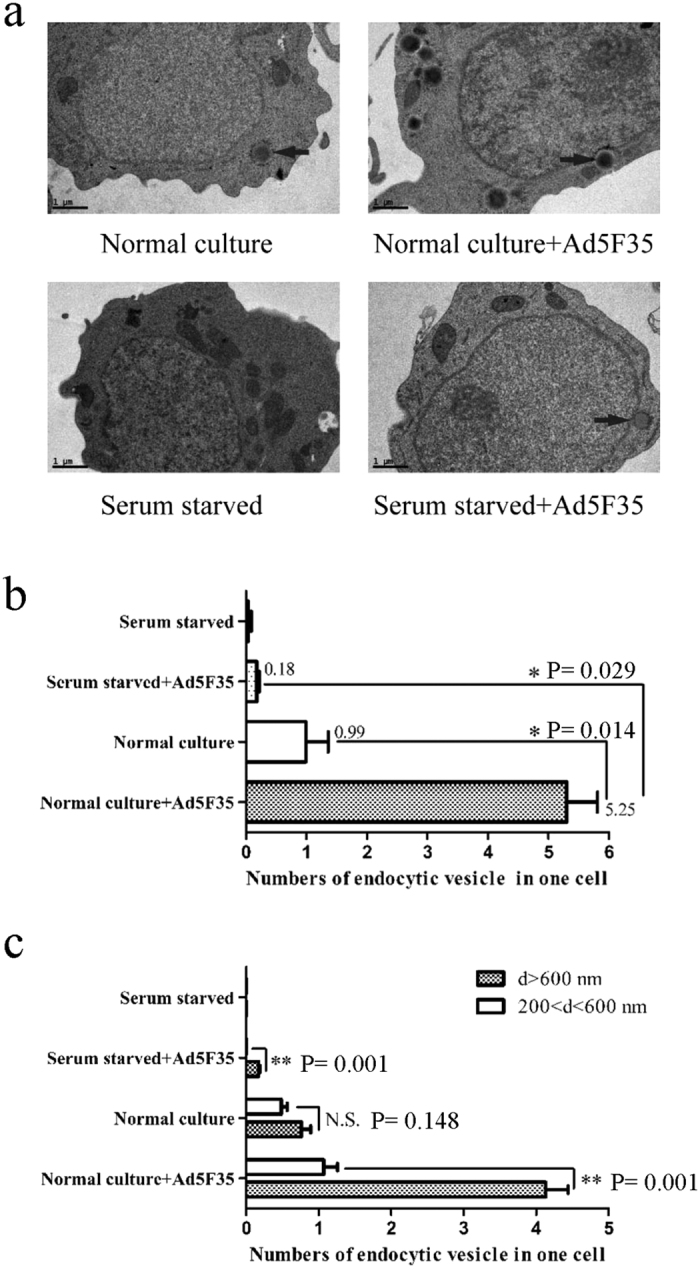
Electron microscopy analysis of endocytic vesicles. (**a**) Untreated or serum-starved Jurkat cells were incubated with Ad5F35 for 50 minutes at 37 °C at an MOI of 100. Arrows indicate endocytic vesicles and the bar represents 1 μm. (**b**) Quantitative analyses of endocytic vesicles in each group. Fifteen micrographs selected randomly were examined in each group and the number per cell was determined. The data are presented as the mean ± standard error of three experiments. (*p < 0.05). (**c**) Vesicles were examined on the basis of two sizes (Vesicle size (VS) >600 nm; 200 < VS <600 nm). Ten micrographs were randomly examined in each group. The number of endocytic vesicle with different size was calculated as described in the Material and Methods. The data are presented as the mean ± standard error of three experiments. (**p < 0.01, N.S. no statistical significance).

Once bound to the cell surface, the adenovirus enters the cell by endocytosis^9^,^10^. Because serum starvation does not affect viral binding to cells, we next analyzed the effect of starvation on viral entry into cells. To this end, adenovirus transport and entry into cells through endocytic vesicles was observed using electron microscopy (Fig. 2a). We found that a number of endocytic vesicles were present in cells from the untreated culture group one hour after viral infection, whereas such endocytic vesicles were absent in the serum-starved group (Fig. 2a). Next, fifteen cells were randomly selected from each group for statistical analysis (Fig. 2b). We observed a significant difference in the number of endocytic vesicles in virus-infected cells from the untreated culture group (5.25/cell) compared with virus-infected cells from the serum starvation group (0.18/cell) and the non-infected, untreated group (0.99/cell) (ANOVA; F = 59.555; df = 3; p-value = 0.029; Serum starved + Ad5F35 group comparing Normal culture + Ad5F35 group; p-value = 0.014; Normal culture group comparing Normal culture + Ad5F35 group). These results suggest that Ad5F35 enters Jurkat cells via endocytosis, which is inhibited by serum starvation. We next analyzed the sizes of endocytic vesicles in randomly selected cells from each group and found that large (greater than 600 nm in diameter) endocytic vesicles were predominant in cells after Ad5F35 infection of both untreated and serum-starved cultures (Fig. 2c). The size of endocytic vesicles formed in target cells after infection with a chimeric adenovirus (HAdV2/BAdV4) is associated with the time of viral infection^11^. Moreover, giant endocytic vesicles gradually predominate as time of infection increases^11^, consistent with our own observations. Macropinocytosis mediated by large vesicles (0.6–5 μm in diameter) is reportedly an entry mechanism of Ad5F35 into B lymphocytes with the formation of large endocytic vesicles being dependent on cell type^12^; however, little is known about the mechanism and biological significance of Ad5F35-induced large endocytic vesicles.

### Changes in cell membrane fluidity alter the efficiency of infection with Ad5F35

Cell membrane fluidity has been shown to play an important role in endocytosis^13^. Thus, we next examined whether serum starvation affects cell membrane fluidity using fluorescence recovery after photobleaching (FRAP). Two typical kinetic parameters can be discerned from quantitative studies using FRAP: the mobile fraction (Mf), which is the fraction of fluorescent dyes that can diffuse into the bleached region during the time course of the experiment, and the diffusion constant (D), which is a measure of the rate of dye movement.

**Figure 3:**
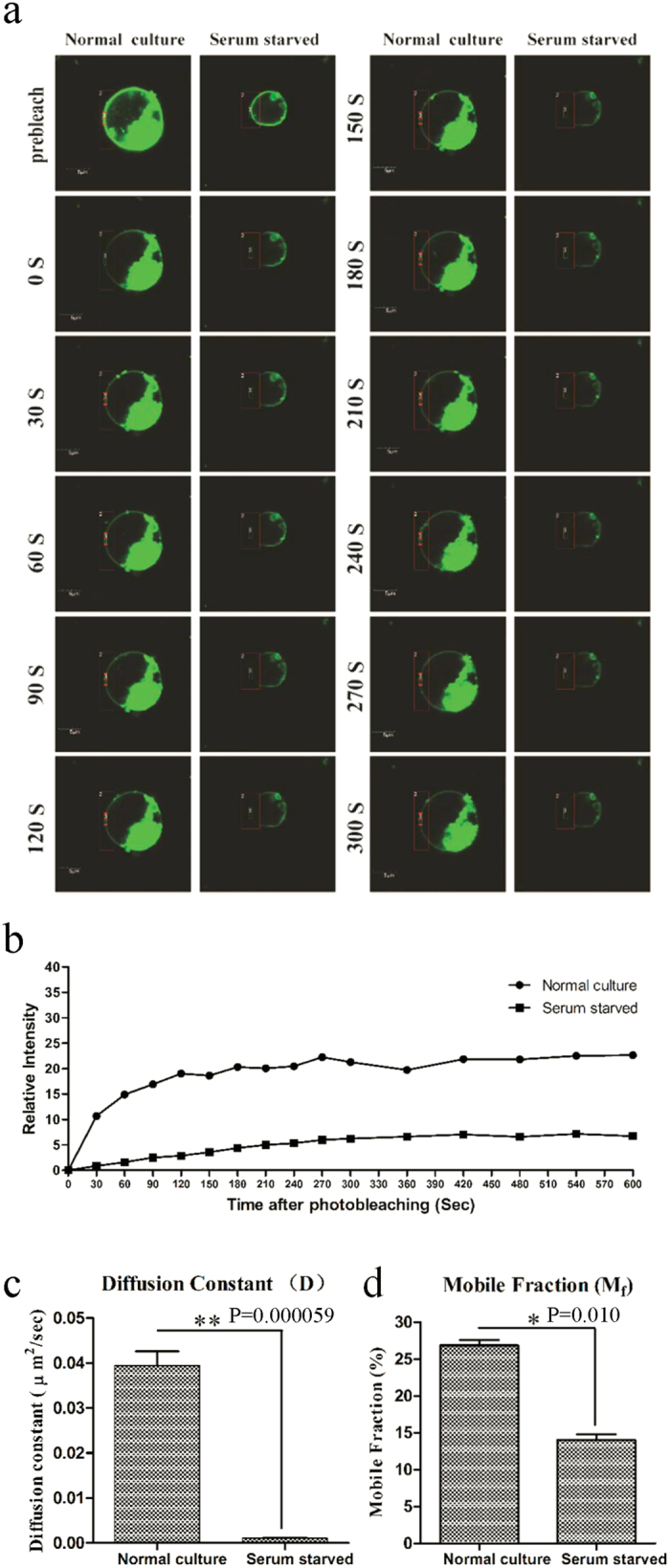
Serum starvation regulates plasma membrane fluidity. Plasma membrane fluidity was monitored in serum-starved Jurkat cells and compared to untreated cells using FRAP analysis of the lateral diffusion of incorporated DiO. After background normalization, the fluorescence was plotted over time to generate fluorescence recovery curves. Analysis of the curves using the formula 1: 
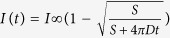
 resulted in two values, the mobile fraction and the diffusion constant. (**a**) Typical fluorescence recovery images and (**b**) recovery curves are shown. Statistical analyses of two measures, (**c**) diffusion constant and (**d**) mobile fraction revealed the speed of fluorescence recovery and degree, respectively. (*p < 0.05, **p < 0.01).

Typical fluorescence recovery images are shown in Fig. 3a. The slope of the FRAP curve is markedly higher for the untreated culture relative to the serum-starved culture indicating decreased membrane diffusion in response to serum starvation (Fig. 3b). Notably, as shown in Fig. 3c, there was a significant difference in the diffusion constant (D) after treatment (untreated culture vs. serum starvation = 0.039 μm^2^/s vs. 0.001 μm^2^/s, respectively) (Independent-Samples T Test; t = 17.781; p-value = 0.000059). Figure 3d shows that the mobile fraction (Mf) was also significantly different before and after treatment (untreated culture vs. serum starvation = 26.8% vs. 14%, respectively) (Independent-Samples T Test; t = 4.6; p-value = 0.010). Taken together, these data suggest that serum starvation significantly reduces cell membrane fluidity in Jurkat cells.

**Figure 4:**
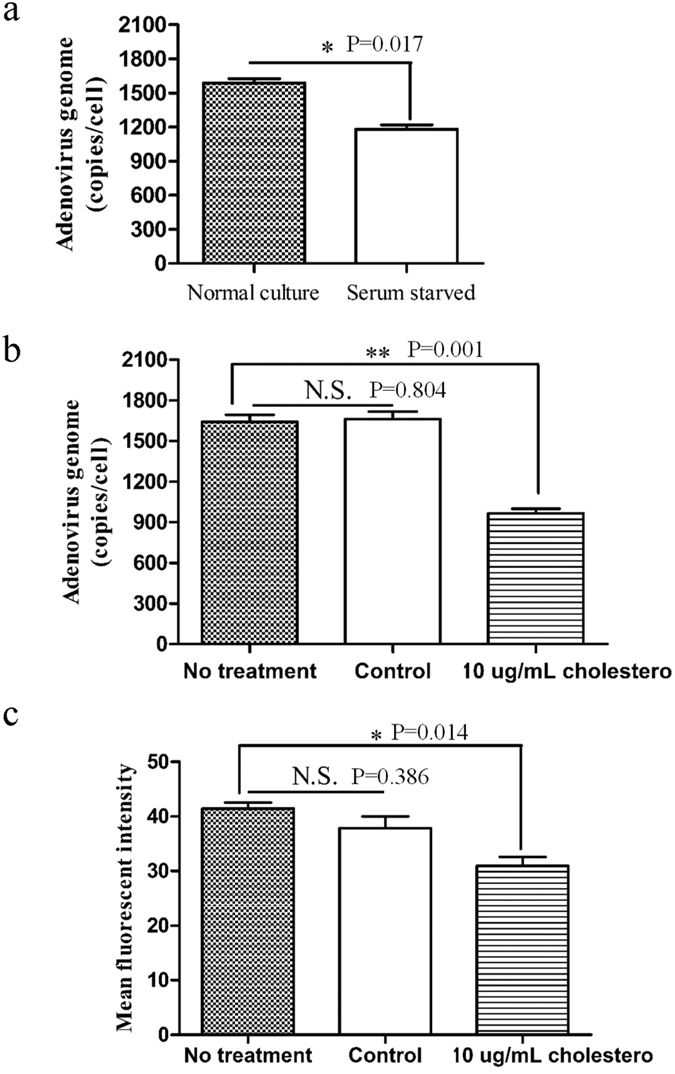
Quantitative evaluation of Ad5F35 genome internalization into Jurkat cells. (**a**) Jurkat cells were serum starved or left untreated followed by infection with Ad5F35 at 37 °C for 60 min at an MOI of 100. Virus-containing medium was removed and cells were washed twice with PBS prior to being harvested and having DNA extracted. The number of viral genomes was quantitated by real-time fluorescent PCR using primers specific for the fiber sequence and compared to an internal standard (cellular GAPDH gene). The data are presented as the mean ± standard error of three experiments (*p < 0.05). (**b**) Jurkat cells were cultured in the presence/absence of free cholesterol (10 μg/mL) or ethanol (control) for 24 h. The cells were then infected with Ad5F35 at 37 °C for 60 min at an MOI of 100. Cells were washed twice with PBS prior to being harvested and having DNA extracted. The number of viral genomes was quantitated by real-time fluorescent PCR as above. The data are presented as the mean ± standard error of three experiments (**p < 0.01, N.S. no statistical significance). (**c**) Jurkat cells were cultured in the presence/absence of free cholesterol (10 μg/mL) or ethanol (control) for 24 h. The cells were then infected with Ad5F35-GFP at 37 °C for 6 h at an MOI of 100. Cells were washed and incubated at 37 °C for another 36 h. The cells were then harvested and analyzed by flow cytometry. The data are presented as the mean ± standard error of three experiments. (*p < 0.05, N.S. no statistical significance).

Next, we verified whether the differences observed in membrane fluidity upon serum starvation affect viral entry. The amount of virus entering target cells was determined by fluorescence quantitative PCR. Consistent with the observations made using electron microscopy (Fig. 2), serum starvation was found to lead to a reduction in viral entry. In this regard, our results showed that the amount of virus entering cells in the serum-starved group was 1,181 ± 36.5 copies/cell, as compared to 1,588 ± 37.5 copies/cell in the untreated culture (Fig. 4a) (Independent-Samples T Test; t = 7.656; p-value = 0.017). Cholesterol is a critical factor affecting cell membrane fluidity and the addition of exogenous cholesterol results in lower membrane fluidity^14^,^15^. Therefore, cells were cultured in media containing 10 μg/mL cholesterol for 24 hours and then infected with virus. In the untreated group there were 1,642 ± 50.7 viral copies/cell, in the control group there were 1,661 ± 56.3 copies/cell, and in the cholesterol-treated group 965 ± 35.4 copies/cell (Fig. 4b) (ANOVA; F = 55.078; df = 2; p-value = 0.001; No treatment group comparing 10 μg/mL cholesterol group). These data demonstrate that a reduction in cell membrane fluidity results in reduced viral entry. Notably, the percent of GFP-positive cells (~50%) did not significantly change after 48 hours of Ad5F35-GFP infection in the cholesterol-treated culture compared with the untreated culture and the control culture (data not shown). Only the mean fluorescence intensity (MFI) decreased (from 42 to 30.6) suggesting that cholesterol-induced changes in cell membrane fluidity affect the amount of virus entering a single cell but may not affect the overall efficiency of viral infection (Fig. 4c) (ANOVA; F = 9.847; df = 2; p-value = 0.014; No treatment group comparing 10 μg/mL cholesterol group). Thus, these results demonstrate that serum starvation results in decreased cell membrane fluidity thereby reducing the amount of virus entering cells.

### Alteration of Ad5F35 endosomal membranes lysis efficiency across states

After an adenovirus enters a cell via endocytosis, the virus will then cross into the endosome^16^. An ATP-driven proton pump within the endosomal membrane pumps H^+^ into the endosome to maintain an acidic environment that causes partial capsid proteins to disassemble at low pH levels, inducing the lysis of the endosomal membrane and subsequent viral escape into the cytoplasm^17^. As described above, our experimental data confirm that serum starvation induces changes in cell membrane fluidity (Fig. 3). Thus, given that cell membrane-derived endocytic vesicles and the endosomal membrane fuse during endocytosis, we hypothesized that serum starvation may also affect viral lysis of the endosome.

**Figure 5:**
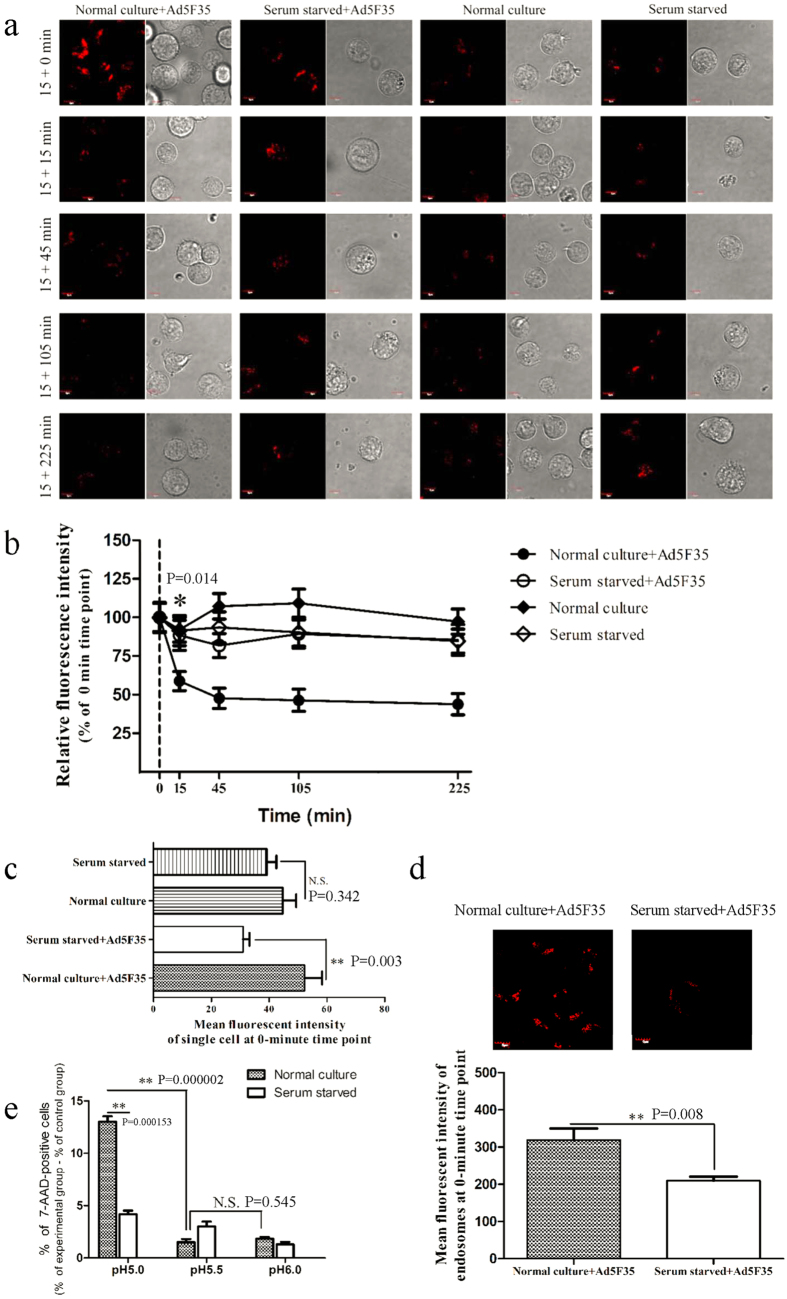
The ability of Ad5F35 to induce endosomal lysis under various physiological states. (**a**) Jurkat cells were prepared under conditions of normal culture or serum starvation for 72 h followed by the addition of pHrodo Red-coupled dextran. Cells were cultured with or without Ad5F35-GFP and incubated at 37 °C for 15 min. The cells were washed with PBS. Nextly, cells were incubated for different durations (0, 15, 45, 105, and 225 min) and red fluorescence was observed by laser scanning confocal microscopy. (**b**) In each experimental group, 30 cells were randomly selected from a field of view, and ten endosomes were randomly selected from the red fluorescence regions in each cell. The MFI at 0 min was recorded as 100%, and changes in the relative fluorescence intensity at other time points were examined. The data are presented as the mean ± standard error of three experiments (*p < 0.05, Normal culture + Ad5F35 group comparing Serum starved + Ad5F35 group). (**c**) For each experimental group, 30 cells were randomly selected from the field of view. The whole-cell MFI at 0 min was calculated for each group. The data are presented as the mean ± standard error of three experiments (**p < 0.01, N.S. no statistical significance). (**d**) In each experimental group, 30 cells were randomly selected from the field of view, and ten endosomes were randomly chosen from the red fluorescent regions in each cell. The MFI of the endosomes at 0 min were calculated for each group. The data are presented as the mean ± standard error of three experiments (**p < 0.01). (**e**) Jurkat cells were prepared under conditions of normal culture or serum starvation for 72 h. Cells were then transferred into buffers with different pH values. The Ad5F35-GFP was added to the cell suspensions and incubated at 37 °C for 1 h. The DNA dye 7-AAD was added, and the percentage of lysed cells was calculated by measuring the percentage of 7-AAD-positive cells. The data are presented as the mean ± standard error of three experiments (N.S. no statistical significance, **p < 0.01).

pHrodo Red is a fluorescent dye that is widely used to study cell endocytosis as the fluorescence intensity of pHrodo is inversely related to pH^18^. Therefore, we incubated target cells with pHrodo Red-coupled dextran and virus and monitored changes in the relative fluorescence intensity as a function of time in order to assess viral lysis of the endosome (Fig. 5a,b). As shown in Fig. 5b, we observed that compared to the mean fluorescence intensity of endosomes at 0-minute time point (set to 100%), the fluorescence intensity was significantly reduced in the untreated culture at 15-minute time point (to approximately 55%), indicating the induction of endosomal lysis (ANOVA; F = 3.428; df = 3; p-value = 0.014; Serum starved + Ad5F35 group comparing Normal culture + Ad5F35 group). In contrast, the fluorescence intensity in the serum-starved culture was only slightly reduced at the 15-minute time point, indicating a weakened ability of the virus to induce endosomal lysis under these conditions. Thereafter, no significant changes in the percentage of fluorescence intensity were observed over time. Collectively, these results indicate that Ad5F35 induces the lysis of the endosome within 30 minutes after viral infection, an effect that is suppressed in serum-starved cells. To analyze the MFI per cell, 30 cells were randomly selected and the whole-cell MFI was calculated at 0-minute time point (Fig. 5c). The MFI in the serum starvation group was lower than that of the untreated culture group (ANOVA; F = 4.000; df = 3; p-value = 0.003; Serum starved + Ad5F35 group comparing Normal culture + Ad5F35 group). This suggests that the virus has a greater ability to enter cells in the untreated culture relative to the serum-starved culture, consistent with electron microscopy observations (Fig. 2).

### Alteration of the ability of Ad5F35 viral capsid proteins to disassemble in the endosome

**Figure 6:**
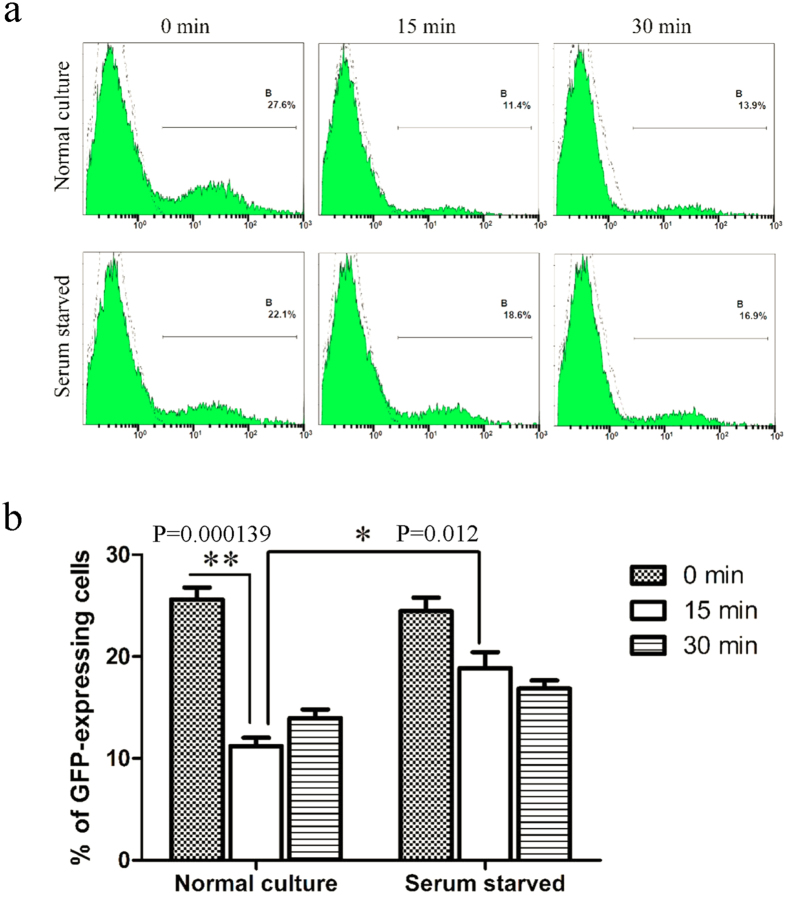
Infectivity of Ad5F35 vectors on HeLa cells after their recovery at different time points from infected Jurkat cells by freeze-thawing. (**a**) Determination of infectivity of Ad5F35 from infected Jurkat cells after freeze-thawing by flow cytometry. Jurkat cells treated with serum starvation or normal culture were infected on ice with Ad5F35-GFP at MOI of 100. Unattached virus particles were then removed and cells were transferred to 37 °C for different times and were subjected to four freeze-thaw cycles to release cell-associated virus particles. In the control settings (0 min) cells with attached viruses were lysed without incubation at 37 °C. Cell-associated virus particles were obtained and applied on HeLa cells, and the percentage of GFP-expressing cells was analyzed 48 h later by flow cytometry. (**b**) Percentage of GFP-expressing HeLa cells in different time point. Results represent the mean of three independent experiments with errors bars corresponding to standard error (*p < 0.05, **p < 0.01).

After adenovirus enters the endosome, viral capsid proteins undergo conformational changes and partially disassemble due to the low pH. Preceding viral escape, the fiber is completely detached from the virus particle and other viral capsid proteins (penton base, IIIa, VIII, and IX) successively start to disassemble and detach from the viral particle^19^. After the disassembly of its capsid proteins, the virus loses the ability to re-infect cells. To determine whether serum starvation-induced changes affecting the ability of a virus to induce endosomal membrane lysis are associated with changes in the ability of viral capsid proteins to disassemble, we incubated the Ad5F35-GFP virus with untreated or serum-starved Jurkat cells on ice for one hour, a condition that allows for viral binding but not viral entry. The unbound virus was washed away, and the cells were maintained at 37 °C for various amounts of time (0, 15, and 30 minutes). After the cell suspension was repeatedly frozen and thawed, the supernatant was collected and used to infect HeLa cells. The percentage of GFP-expressing cells at different time points were analyzed by flow cytometry (Fig. 6a). Our results showed that from the 15-minute time point relative to the 0-minute time point the percentage of GFP-positive HeLa cells infected with virus was significantly reduced in the untreated culture (26% to 11%, respectively) (ANOVA; F = 31.493; df = 5; p-value = 0.000139; Normal culture-0 min group comparing Normal culture-15 min group). Notably, the reduction was less significant in the serum-starved group (24% to 19%, respectively) (Fig. 6b). These results indicate that the ability of viral capsid proteins to disassemble was markedly reduced in serum-starved cells, resulting in a decreased ability to induce lysis of the endosomal membrane.

## Discussion

Adoptive transfer of genetically-modified T cells is a promising approach for the therapy of both human malignancy and viral infections^20^,^21^. To date, gene transfer to T cells has mainly focused on viral vector systems^4^,^22^. Therefore, a viral vector system that yields a high infection efficiency is key to the T-cell therapy. Due to its ability to efficiently infect lymphocytes, the chimeric adenovirus Ad5F35 is a valuable vector system for T-cell therapy.

We and others found that stimulation of T cells results in increased Ad5F35 infection efficiency without significant changes in viral receptor expression^4^. Given the complexity of adenovirus infection pathway, factors besides receptor expression affect the efficiency of Ad5F35 infection of T cells. To clarify the as yet unclear underlying mechanisms will help further the clinical application of genetically-modified T cells for immunotherapy.

PBMCs are heterogeneous and the purification of T cells often results in their stimulation. Hence, we selected the Jurkat (leukemic T cell line) for use as a model in our experiments. Given the activated status of Jurkat cells, the serum starvation method was used to inversely induce changes in the physiological state of Jurkat cells, which successfully replicated the observed *in vitro* phenomenon of altered efficiency of viral infection without changes in viral receptor expression.

The classical pathway of adenovirus infection and intracellular transport is generally divided into five parts: binding, entry, escape, translocation, and nuclear import^23^. Serum starvation negatively impacts adenoviral infection; however, it does not result in any significant change in the expression of classical viral entry receptor molecules (CD46 and integrin αVβ3) or of newly-identified receptors (CD80 and CD86), nor in the amount of virus bound per cell (Fig. 1). Thus, this indicates that previously reported alterations in expression of viral receptors is not the only determinant that can affect the efficiency of Ad5F35 infection.

During the process of viral entry we found that serum starvation strongly affects the transport of virus into cells via endocytosis (Fig. 2). Several studies have shown that changes in the expression of integrin molecules play an important role in mediating viral endocytosis into target cells^24^,^25^,^26^. However, our experiments demonstrate very low expression levels of integrin αVβ3, with no significant changes in expression after serum starvation (Fig. 1g,h) suggesting that other factors affect endocytosis. Ben-Dov *et al.* found that proton-induced endocytosis is directly correlated with cell membrane fluidity^13^. Therefore, we analyzed the effect of serum starvation on cell membrane fluidity and found that both the mobile fraction (*Mf*) and the diffusion constant (*D*) were significantly reduced after serum starvation (Fig. 3c,d). Furthermore, following cholesterol treatment, cell membrane fluidity is reduced resulting in a decrease in the amount of Ad5F35 entering cells (Fig. 4b). Together, these results suggest that serum starvation alters the fluidity of the cell membrane and thereby affects the endocytic entry of a virus into a cell.

Lai *et al.* found that dividing cells have higher membrane fluidities than resting cells^27^. An *in vitro* study by Fukaya *et al.* indicated that the cell membrane fluidity of rapidly proliferating rat nerve cells derived from young rats is higher than that of slowly proliferating nerve cells from older rats^28^. These results indicate that cell membrane fluidity is closely related to the capacity of a cell to proliferate. In our experiments (Table S1), cell proliferation slowed after serum starvation. Therefore, we speculated that the reduction in cell membrane fluidity after serum starvation was associated with the slowed cell proliferation.

The ability of a virus to lyse the endosomal membrane, a process necessary for viral escape, is reduced after serum starvation (Fig. 5). Further, the degree of adenovirus capsid protein disassembly in the endosome is a critical factor affecting viral lysis of the endosomal membrane and subsequent viral escape into the cytoplasm^29^,^30^. We found that the disassembly of capsid proteins such as fiber/penton is inhibited after serum starvation (Fig. 6) indicating that serum starvation affects the disassembly of viral capsid proteins and, therefore, also affects viral lysis of the endosomal membrane.

*In vitro* analysis of adenoviral cell membrane lysis is often used to simulate the process of viral lysis of the endosome^31^,^32^,^33^. In this study, we analyzed Ad5F35-induced lysis of the cell membranes of Jurkat cells cultured *in vitro* and found no significant lysis of Jurkat cell membranes at pH 5.5 or 6.0 (Fig. 5e), suggesting that a lower pH level is more favorable for the Ad5F35-induced lysis of the cell membrane. Previous studies also showed that endosomal pH has a significant effect on the disassembly of capsid proteins, with lower pH values being more conducive to disassembly^34^,^35^. To investigate the effects of serum starvation on endosomal pH, we incubated pHrodo Red-coupled dextran and virus with cells for 15 minutes and then randomly selected ten endosomes from the red fluorescent areas of each of 30 cells and calculated the MFI. As shown in Fig. 5d, the MFI was lower in serum-starved cells than in untreated cells, indicating a higher endosomal pH (Independent-Samples T Test; t = 3.337; p-value = 0.008). Thus, we inferred that serum starvation leads to an increase in pH in the endosome and a reduction in the ability of viral capsid proteins to disassemble, thus inhibiting viral lysis of the endosome and subsequent viral escape.

**Figure 7:**
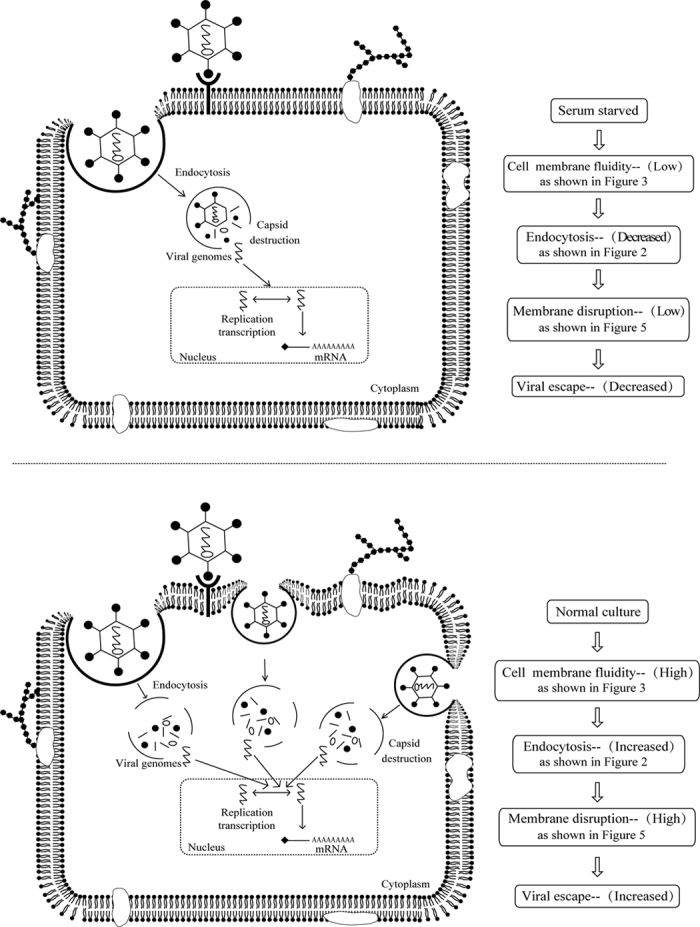
A schematic model showing how the infection of Ad5F35 is affected by the changes of cell physiological state.

In summary, our experimental results indicate that in addition to the membrane receptor, the infection efficiency of Ad5F35 was also affected by other factors (Fig. 7) including: 1) changes in the efficiency of virus-induced endocytosis, 2) changes in cell membrane fluidity affecting viral entry into cells, and 3) differences in the ability of viral capsid proteins to disassemble (e.g., fiber/penton) affecting virus-induced endosomal lysis. These findings suggest that stimulating T cells to induce the changes in the physiological state could increase Ad5F35 infection efficiency, which is of great value to the development of adoptive immunotherapy based on the genetic modification of T cells.

## Methods

### Cell Lines and PBMCs

PBMCs were isolated from the whole blood of healthy volunteers with Ficoll-PaqueTM PLUS (GE Healthcare, Sweden) according to the manufacturer’s protocol. All participants signed an informed consent document. The protocols used for human studies were approved by the ethics committee of Guangdong Pharmaceutical University and the methods were carried out in accordance with the approved guidelines. The PBMCs were suspended at 1 × 10^6^ cells/mL in RPMI-1640 medium supplemented with 10% fetal bovine serum, 2 mM L-glutamine (Gibco BRL), 100 U/mL penicillin, 100 μg/mL streptomycin, and incubated at 37 °C under 5% CO_2_.

Stimulation of PBMCs was performed with 1000 U/mL interferon γ (R&D Systems, Tokyo, Japan) on day 0, with 25 ng/mL monoclonal antibody (MoAb) anti-CD3 (OKT 3; R&D Systems) and 300 IU/mL recombinant IL-2 (R&D Systems) on day 1, and with 1000 IU/mL IL-2 every 3 to 5 days thereafter for a total of 15 days.

The Jurkat/E6-1 (T cell leukaemia) and HEK293 (human embryonic kidney) cell lines were purchased from the American Type Culture Collection (ATCC). Jurkat cells were cultured in RPMI-1640 medium (Gibco, Grand Island, NY), and the HEK293 cell line was maintained in Dulbecco’s modified Eagle medium (DMEM; Gibco, Grand Island, NY). The cells were grown in their specific medium supplemented with 10% foetal calf serum or 1% foetal calf serum (FCS; HyClone, Logan, UT), 100 μg/mL streptomycin and 100 U/mL penicillin at 37 °C in a humidified incubator with 5% CO_2_. The adenoviral shuttle plasmid pDC-315 and the genomic plasmid were purchased from Microbix (Toronto, Ontario, Canada).

### Construction of Adenoviral Vectors

The Ad5F35 constructs were generated as previously described^36^. The virus preparations were quantitated using OD_260_ measurements. The viral titres were determined using a plaque-forming assay in HEK293 cells, and the virus was purified as previously described^36^.

### Flow Cytometry Analysis

Fluorescent-labelled antibodies for flow cytometry were purchased from eBioscience (San Diego, CA). The expression of CD46 and β3 integrin in the T cell lines and primary T cells were analysed using flow cytometry with anti-human CD46 and anti-human CD61 (β3) antibodies conjugated to phycoerythrin (PE) and fluorescein isothiocyanate (FITC), respectively. The data were acquired using an Epics-XL flow cytometer (Beckman Coulter, Inc. Miami, FL) and analysed using EXPO32 v1.2 software.

The analysis of adenoviral transduction was performed using a previously published protocol^4^. The proportion of T cell lines transduced by adenoviral gene transfer were quantified using flow cytometry.

### Quantitative Analysis of Ad Genome Delivery

Jurkat cells were infected with Ad5F35 at a dose of 100 MOI for 50 min. The cells were subsequently washed three times with serum-free medium.Total cellular DNA was extracted using the QIAamp blood kit (QUIAGEN,Inc.,Santa Clara,CA). The Ad fiber gene and GAPDH gene were amplified and quantified by real-time PCR. For Ad5F35 analysis, the sense primer (GCCTTTTCTTACTCCTCCCTTTGT) and antisense primer (ATCCGCCTGTGGTTGTTAGTG) specific for the fiber gene,was used. For human GAPDH detection, the sense primer (CCCTTTTGTAGGAGGGACTTAGAGA) and antisense primer (CATCAAACTCAAAGGGCAGGA) were used. Samples were amplified for 40 cycles in a Perkin–Elmer 7700 sequenced detection system with continuous monitoring of fluorescence. Data were processed by the SDS 1.6 soft ware package (Perkin-Elmer).

### Electron Microscopic Analysis

2 × 10^6^ Jurkat cells cultured 72 h with 10% serum or 1% serum were infected with Ad5F35 at MOI 100 for 50 minutes at 37 °C,The cells were then washed with cold PBS and fixed in 2% paraformaldehyde–1.5% glutaraldehyde in 0.1 mol/L sodium cacodylate bufer, pH 7.4, for 2 hours at 4 °C. The cells were washed three times in sodium cacodylate before post fixation in 2% osmium tetroxide for 1 hour. They were then embedded in Alradite and ultrathin sections were counterstained with 2% uranylacetate. Observations were made on a transmission electron microscope Zeiss EM 902. Jurkat cells cultured with 10% serum or 1% serum after 72 h were used as control.

### Membrane Fluidity-FRAP Method

As described previously^28^, Jurkat cells cultured 72 h with 10% serum or 1% serum were loaded with the dye DIO, which is known to incorporate into membranes, for 5 min at room temperature. Cells were washed with RPMI-1640 medium and fixed with low-melting point agarose. Fluorescence recovery after photobleaching (FRAP) was measured with an oil-immersion objective in a laser-scanning microscope (Olympus). A small area of the labelled membrane was selected, and its fluorescence was photobleached with 100% laser pulses (488 nm). Complete or near-complete photobleaching was achieved after 15 scans with a total duration of 30s. The fluorescence intensity was recorded at an excitation wavelength of 488 nm and an emission wavelength of 501 nm at 1.5 μm axial resolution every 30s over a period of 5 min before and after photobleaching. After background normalization, the fluorescence was plotted over time to generate fluorescence recovery curves.

As described previously^28^, analysis of the curves using the formula 1: 
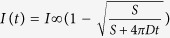
, where I(t) is the relative fluorescence intensity at the region of interest, *S* the spot area in μm^2^ and *I*_∞_ is the estimated infinite recovery level of the fluorescence intensity. The non-linear least squares fitting tool in Origin (Version 6, Microcal, Northampton, MA) was used to estimate the best non-linear curve using the above equation.

Recovery of the bleached region was characterized by two parameters: mobile fraction (*Mf*) and diffusion constant (*D*). *Mf* was calculated by the following formula 2: 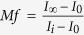, where *I*_*0*_ is the minimum fluorescence intensity after bleaching and *I*_*i*_ is the initial intensity before bleaching. In the present study, *Mf* was identical to *I*_∞_ because the fluorescence intensity was expressed as the relative value, *I*_*0*_ and *I*_*i*_ were set as 0 and 1, respectively. *Mf* represents the fraction of fluorescent molecules available to move into the bleached area over the time course of the experiment, whereas *D* provides a measure of how fast recovery occurs.

### Ad-induced Membrane Lysis Assay

Ad-induced cell membrane lysis was assayed by measuring the percentage of lysed cells. 1 × 10^6^ Jurkat cells cultured 72 h with 10% serum or 1% serum were washed once with RPMI-1640 medium and incubated at 4 °C for 1 h with Ad5F35 (MOI = 100). After virus binding, the cell samples were washed once with permeability buffer, prepared by titrating morpholineethanesulfonic acid (MES)-buffered saline (100 Mm MES [pH5.0] containing 0.9% NaCl, 0.2% BSA, 1 mM CaCl_2_,1 mM MgCl_2_, and 50 mM NaN3) with HEPES-buffered saline (100 mM HEPES [pH 7.5] with the same supplements) to achieve the desired pH. The cells were incubated at 37 °C for 1 h. Virus binding was performed in the presence of sodium azide to eliminate cytosolic ATP and prevent endocytosis. After incubation, 7-amino actinomycin D (7-AAD) was added to test membrane integrity. Following incubation, cells were washed and analyzed by flow cytometry. The percentage of lysed cells was calculated by measuring the percentage of 7-AAD-positive cells.

pHrodo™ Red dextran possesses a pH-sensitive fluorescence emission that increases in intensity with increasing acidity and is essentially non-fluorescent in the extracellular environment and the near-neutral pH of the cytoplasm; However, upon internalization, the acidic environment of the endosomes elicits a bright, red-fluorescent signal from this dextran conjugate. To analyze the Ad-induced endosome membrane lysis, 1 × 10^6^ Jurkat cells cultured 72 h with 10% serum or 1% serum were infected with Ad5F35 containing pHrodo Red dextran at MOI 100 for 15 minutes at 37 °C. After the supernatant was discarded, the cells were washed with PBS. Nextly, cells were incubated for different durations (0, 15, 45, 105, and 225 min) and red fluorescence was observed by laser scanning confocal microscopy. To calculate the mean fluorescence intensity (MFI), 30 cells were randomly selected from a field of view, and ten endosomes were randomly selected from the red fluorescence regions in each cell. The MFI at different time points was calculated using laser scanning confocal microscopy software.

### Statistical Analysis

The Data were represented as the mean ± standard error (SE) of more than three separate experiments. The statistical analysis was performed using the Analysis of Variance (ANOVA) test or Independent-Samples T Test. A p-value less than 0.05 was considered to be significant. SPSS 20.0 software (IBM, Armonk, USA) was used for these analyses.

## Additional Information

**How to cite this article**: Zhang, W.-f. *et al.* Influence of cell physiological state on gene delivery to T lymphocytes by chimeric adenovirus Ad5F35. *Sci. Rep.* **6**, 22688; doi: 10.1038/srep22688 (2016).

## Supplementary Material

Supplementary Information
