# Supplementary material for: Influence of cell physiological state on gene delivery to T lymphocytes by chimeric adenovirus Ad5F35
Source: Sci Rep. 2016 Mar 14;6:22688. doi: 10.1038/srep22688 (PMC4789598; doi:10.1038/srep22688)
Supplement: Supplementary Information [file srep22688-s1.pdf]

Supplemental information

Influence of cell physiological state on gene delivery to T lymphocytes by chimeric adenovirus Ad5F35

Wen-feng Zhang[1,2,3], Hong-wei Shao[1,2,3], Feng-lin Wu[1,2,3], Xin Xie[1,2,] Zhu-Ming Li[1,2], Hua-Ben Bo[1,2], Han Shen[1,2], Teng Wang[1,2], Shu-lin Huang[1,2*]

[1]Guangdong Province Key Laboratory for Biotechnology Drug Candidates, Guang dong Pharmaceutical University, Guang zhou, People's Republic of China

[2]School of Biosciences and Biopharmaceutics, Guangdong Pharmaceutical University,

Guang zhou, People's Republic of China

[3]Co-first author

[*]Correspondence:shulhuang@sina.com

Table S1. Influence of serum starvation on cell cycle and cell proliferative activity

Group	G_0/G_1	S	G_2M	viable cell number ($\times 10^5$)
Normal culture	54.1 ± 0.7	19.6 ± 0.5	21.5 ± 0.7	15.1 ± 0.4
Serum starvation	75.5 ± 0.8 *	9.6 ± 0.4	10.5 ± 0.5	6.2 ± 0.3 **

The data are presented as the mean ± standard error of three experiments. The statistical analysis was performed using the t-test. * $p < 0.05$ vs Normal culture group, ** $p < 0.01$ vs Normal culture group.

Supplemental Experimental Procedures

5×10^5 Jurkat cells were cultured with 10% serum or 1% serum for 72 h. Cell number/viability was determined using Cellometer based on the trypan blue exclusion method (Pathak et al, 2011). For cell cycle analysis, cells were harvested and mixed with detergent solution (0.1% Triton, 0.15 M NaCl,0.1 M HCl), centrifuged, resuspended in 50 µl RNase solution (RNaseA 100 µg/ml, 1% trisodium citrate) and incubated at 37 ℃ for 15min. Cells were washed in 0.1 M Tris-HCl pH7.4, and stained for 30min (65 µg/ml propidium iodide). Cell cycle was analyzed by flow cytometry.

Supplemental References

Pathak, S., Goldofsky, E., Vivas, E.X., Bonagura, V.R., & Vambutas, A. IL-1β is overexpressed and aberrantly regulated in corticosteroid nonresponders with autoimmune inner ear disease. *J. Immunol.* **186**, 1870-1879 (2011).
